# Creation And Pilot Testing Of The Confidence To Engage In Physical Therapy In Older Adults (CEPT) Measure

**DOI:** 10.1093/geroni/igab046.3246

**Published:** 2021-12-17

**Authors:** Patricia Bamonti, Rebekah Harris, Jennifer Moye, Jonathan Bean

**Affiliations:** 1 VA Boston Healthcare System, Boston, Massachusetts, United States; 2 VA Boston Healthcare System, VA Boston Healthcare System, Massachusetts, United States

## Abstract

The measurement of self-efficacy is important in physical therapy (PT) settings where patients face barriers and adoption of new behavior is critical for recovery. However, existing measures of exercise self-efficacy do not account for the internal (i.e., fatigue) and external (i.e., scheduling) barriers to self-efficacy observed in older adults. We developed a self-report measure assessing an individual’s confidence to engage in PT despite barriers. Qualitative ratings from patients (N = 75; age M =78.26 ± 11.2; 80% white; 20% African American) enrolled in PT at a Veterans Affairs Medical Center (VAMC) and their physical therapist were used to create a 21-item pool for the Confidence to Engage in Physical Therapy in Older Adults (CEPT). We next evaluated item characteristics and scale reliability and validity of the CEPT in a new sample of patients (N = 19; age M = 81.11± 8.93; 88.8% white, 11.1% African American) enrolled in an outpatient PT program who also received the Geriatric Depression Scale-15 (GDS-15), and the Activities-specific Balance (ABC) Scale. Response choice ranged from 0% (not confident at all) to 100% (highly confident) with higher scores indicating greater self-efficacy. Item analyses indicated adequate response variability across items (M = 55.9 ± 24.80, range 10-85). The scale demonstrated evidence of internal consistency reliability (Cronbach’s alpha) = 0.98. Construct validity was demonstrated by positive association between CEPT and the ABC (r = .74, p < .001) and negative association with GDS-15 (r = -.64, p < .01). The CEPT requires further evaluation with larger sample sizes.

